# Patients want to be heard–loud and clear!

**DOI:** 10.1186/s13054-016-1588-7

**Published:** 2017-01-10

**Authors:** Anna-Liisa Sutt, John F. Fraser

**Affiliations:** 1 Speech Pathology Department, The Prince Charles Hospital, Brisbane, Australia; 2 Critical Care Research Group, The Prince Charles Hospital, Brisbane, Australia; 3 School of Medicine, University of Queensland, Brisbane, Australia

We congratulate ten Hoorn et al. [1] on their systematic review of communication with ICU patients. Their work in defining an algorithm to assist improving communication options for these patients addresses a clear gap in patient-centred care in the ICU. Despite the article giving a good overview of possible communication options for the ventilated ICU patient, we respectfully suggest that the most important communication option is the restoration of the patient’s own voice by enabling airflow through their larynx. This is particularly in the conscious patient cohort—the focus of the review article. We are supported by patient data indicating that verbal communication is the most successful form of communication [2]. Once tracheostomised, a speaking valve (SV) should be considered as the first option for communication as it restores our natural way of communication. Beliefs that cuff deflation required for the restoration of laryngeal function with SV causes atelectasis or would be deleterious in the weaning process have been proven to be unfounded [3]. We currently lack published data on the safe ventilatory parameters for SV use; however, patients in our studies using a SV whilst mechanically ventilated had substantial levels of pressure support and PEEP requirements and were able to communicate using a SV in-line with their mechanical ventilation circuit successfully without any discernible harm to their respiratory function or weaning from the ventilator [3].

Using SVs is common in our cardio-thoracic ICU [4] and may commence on the day of tracheostomy insertion, with patients spending hours, sometimes all their awake hours, being able to talk with the treating teams and loved ones.

Following the success of this work, we now use SVs successfully with patients on veno-arterial extracorporeal membrane oxygenation (VA ECMO), ventricular assist devices and open chest. The difference it makes for the patients to have their own voice, and therefore be active participants in their care, is immeasurable with current tools. Studies elsewhere have also demonstrated benefits of early SV use in the ventilated tracheostomised ICU patient [5]. Alternative communication options should be used only if natural communication is not able to be achieved or as complementary devices when verbal communication is not fully successful. In the most critically ill, weakness frequently limits the use of augmentative and alternative communication boards and teaching complex new skills (i.e. electrolarynx) is fraught with difficulty. We concur with the importance of communication but suggest that before moving to more complex interventions, the larynx must always be considered.

## Abbreviations

ICU: Intensive care unit
PEEP: Positive end expiratory pressure
SV: Speaking valve
